# Economic Impact in the Treatment of Coagulase-Negative Staphylococci Blood Cultures Contamination in a Middle-Income Country

**DOI:** 10.1590/0037-8682-0094-2025

**Published:** 2025-08-08

**Authors:** Priscila Gabriella Carraro Merlos, Gustavo Henrique Loesch, Felipe Francisco Tuon

**Affiliations:** 1 Laboratório de Doenças Infecciosas Emergentes, Faculdade de Medicina, Pontifícia Universidade Católica do Paraná, Curitiba, PR, Brasil.; 2 Florida Christian University, Orlando, Florida, USA.

**Keywords:** Coagulase negative *Staphylococcus*, Blood culture, Contamination, Public health

## Abstract

**Background:**

This study aimed to assess the clinical and economic impacts of blood culture contamination in a hospital situated in a low-middle-income country and the breakeven to implement strategies to avoid unnecessary antimicrobials.

**Methods:**

This economic cross-sectional study was conducted at a tertiary hospital, and 8,072 blood cultures were analyzed. Antibiotic duration and cost were calculated in United States dollars (USD). A simulation with a breakeven curve to determine the balance between antimicrobial costs and time-to-result of coagulase-negative staphylococci in one blood culture (suggestive of contamination) was used to define the breakeven point between the cost of the diagnostic tests or prevention strategies and the balance with antimicrobial expense.

**Results:**

Of the 8,072 blood culture samples collected, the contamination rate was 9.9%. Antimicrobial therapy was initiated in 69.6% of the 682 cases of contamination. The median duration of unnecessary antibiotic use was 7 days. The direct costs totaled USD 83,910 annually, comprising USD 73,970 for unnecessary antimicrobials and USD 9,940 for microbiological tests. The extended length of hospital stay potentially contributed to an additional USD 3.87 million in annual hospital costs.

**Conclusions:**

This study underscores the urgent need for strategies to reduce blood culture contamination, and emphasizes the potential benefits of rapid identification techniques for optimizing patient care and healthcare resource utilization. Addressing this issue is of paramount importance for mitigating unnecessary antibiotic exposure, reducing healthcare costs, and improving patient outcomes.

## INTRODUCTION

Blood culture is a critical laboratory test for diagnosing patients with bacteremia^1^. They are deemed contaminated when pathogens originating from the skin of the patient or healthcare professionals, or from contaminated surfaces, are isolated rather than from the patients' blood. Blood cultures are the most accurate method for detecting bacteremia in patients; however, cultures considered false positives account for up to half of all positive blood cultures in adults. The Clinical Laboratory Standards Institute (CLSI) recommends a blood culture contamination rate of <3%, yet the majority of institutions fail to meet this benchmark, exhibiting rates of 2%-10%^2^. Studies show that blood culture contamination rates are higher in teaching hospitals, exceeding 7%^3^.

Coagulase-negative staphylococci (CNS) are the primary causative agents of false-positive blood cultures^4^. These bacteria are significant etiological agents found in hospital blood cultures, making it challenging to differentiate between a sample contaminated by skin or mucosal CNS and true bacteremia, whether or not it is related to venous catheter use^5^. According to the Center for Disease Control and Prevention (CDC) criteria, blood culture contamination is defined as the presence of skin contaminant microorganisms (coagulase-negative staphylococci, *Corynebacterium spp*., *Micrococcus spp*., *Bacillus spp*., alpha or gamma hemolytic *Streptococcus*, *Propionibacteria spp*.) with growth beyond 48 h of incubation or isolation in only one blood culture (paired collections) of any of the aforementioned pathogens^6^. Therefore, in samples analyzed with CNS, it is mandatory for the pathogen to be present in two or more blood cultures and must be linked to a compatible infection clinic, thereby minimizing the indiscriminate use of antimicrobials (ATM).

Contamination of blood cultures with non-pathogenic microorganisms such as skin commensals leads to false-positive results and subsequent unnecessary and potentially harmful interventions for patients. The consequences of treating false-positive blood cultures include an increase in hospitalization time, inadvertent removal of invasive devices, and inadequate administration of ATMs such as vancomycin and, less commonly oxacillin^5^. The use of ATM in patients with contaminated blood culture results is 39% higher than in patients with negative cultures^7^. Similar results were demonstrated by Souvenier et al. and Lee et al., showing that 41% of contaminated blood cultures were treated, with 34% receiving vancomycin unnecessarily^8^. ATM use poses risks, including drug interactions, allergic reactions, alterations in intestinal microbiota with the emergence of *Clostridioides difficile* infection, and development of antimicrobial resistance. However, these effects are difficult to measure and quantify in a study^9^.

A retrospective study conducted in Ireland in 2007 showed that the hospitalization time of patients with contaminated blood cultures increased by approximately 5.4 days, resulting in an increase in hospital bill costs of United States dollars (USD) 7,502 dollars per patient. The same study reported a laboratory cost per patient of USD 61^10^. Little Jr. reported an increase of USD 4,100 in the total hospitalization cost of these patients, an increase of USD 700 in the antimicrobial cost, and an increase of USD 330 in laboratory costs^11^. The best approach to avoid misuse of ATM after contamination is the rapid identification of bacteria using molecular methods, considering the possibility of identification within 24 hours against 4 to 5 days using conventional microbiology.

Given the consistent data in the literature demonstrating the financial impact of a contaminated blood culture, there is a universal consensus that contaminated blood cultures are costly and have significant unintended consequences for patients. Considering the progressive increase in healthcare costs in general, the economic side in cases of false positive blood culture results must be addressed. Thus, professionals responsible for blood collection must be aware of these factors when performing blood culture, as various undesirable factors can permeate blood collection and interfere, directly or indirectly, with the test results and consequently with patient treatment and the management of hospital resources. This study aimed to assess the clinical and economic impacts of blood culture contamination in a hospital situated in a low-to-middle-income country using a laboratory approach with rapid etiological identification tests through simulation.

## METHODS

### Study Design

This was a descriptive, economic, cross-sectional study using the database of a public tertiary general hospital with 279 beds located in Joinville, southern Brazil, as a reference for internal medicine and surgery.

### Study Population

All blood cultures collected from hospitalized patients between July 1, 2022, and June 30, 2023, were evaluated. The project was approved by the Research Ethics Committee (REC) of the Hospital Regional Hans Dieter Schmidt (number 70077523.2.0000.5363). No sample size calculation was performed as all samples within the period were screened for inclusion.

### Inclusion Criteria

Peripheral blood cultures obtained from patients over 18 years old and hospitalized were included. Patients with positive blood cultures who died before the definitive culture result and patients with other infections that could justify antimicrobial use, other than bloodstream infection (BSI), were excluded.

BSI was defined as the recovery of a pathogen from a blood culture (a single blood culture for an organism not commonly present on the skin, and two or more blood cultures for organisms commonly present on the skin). The infection cannot be related to any other infection the patient might have and must not have been present or incubating when the patient was admitted to the facility^6^. The presence of specific commensal organisms cultivated from a single blood culture set from one or more blood culture series was defined as contamination^12^. For analysis purposes, only blood cultures contaminated by CNS were included, and other pathogens considered contaminants were excluded^13^.

### Data Collection and Study Variables

Epidemiological data including age and sex were evaluated. Other data included the number of blood cultures collected, use or nonuse of antibiotics, antibiotic used, duration of antibiotic therapy, and whether a change occurred after the blood culture result. The length of hospital stay (LOS) and in-hospital mortality were analyzed. Although outcome variables such as LOS and mortality were relevant, they were considered secondary in this study because the main variable was the cost attributed to unnecessary antibiotic treatment in cases of blood culture contamination.

Medical records of patients with positive blood cultures were reviewed to determine whether the prescribed antibiotics had any indications other than BSI. The duration of antibiotic use and costs were calculated based on the CEMED table, which regulates the maximum drug costs in Brazil, with the values converted into USD using the average exchange rate during the study period. The costs for microbiological tests included those for culture plates, identification kits, and susceptibility test kits according to the brand and model available during the study period. One simulation was performed using antibiotic consumption (in USD) and a breakeven curve to determine the balance between ATM costs and time-to-result of CNS in one blood culture (suggestive of contamination) to define the breakpoint between the cost of the diagnostic test and the balance with ATM reduction costs.

### Statistical Analysis

The continuous variables were expressed was mean±standard deviation or median (interquartile range 25%-75%) according with normality test (Kolmogorov-Smirnov test). Qualitative variables were expressed as frequencies and percentages. In the bivariate analysis, *P*-values were calculated using the χ2 or Fisher’s exact test for categorical variables and Student’s t-test or Mann-Whitney test for continuous variables. The groups were considered statistically different if the P-value was < 0.05. 

## RESULTS

**FIGURE 1:**
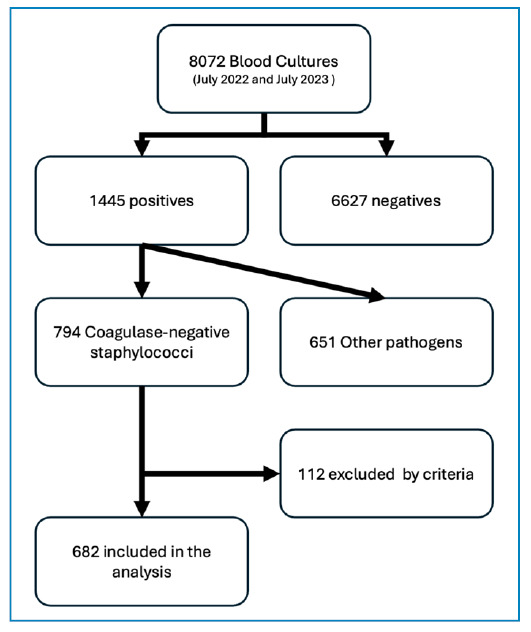
Flowchart with description of the blood culture samples included in the study.

A total of 8072 blood culture samples were collected from all sectors of the institution, of which 1445 (18%) tested positive. Among these positive samples, 794 were identified as coagulase-negative staphylococci (CNS), consistent with contamination (CNS-C), representing 55% of the positive samples and 9.9% of the total collected samples (Table 1). Following the exclusion criteria, 682 patients with CNSC, 583 patients with bloodstream infections (BSI), and 1667 patients with negative blood cultures were excluded (Figure 1).

**TABLE 1:** Clinical Epidemiological Profile of 682 Patients with coagulase-negative staphylococci blood culture contamination vs. 583 patients with bloodstream infection.

Variable	Contamination		Bloodstream infection		P value
	N (682)	Mean/Median or %	N (583)	Mean/Median or %	
Age (mean±SD)	682	65.1±16.1	583	61.3±18.4	P<0.05
Sex (male)	365	53.5	340	58.2	P=0.11
Samples collected (mean±SD)	682	1.5±0.5	583	2.3±0.7	P=0.08
Empirical ATM after BC collect	474	69.6%	503	86.3%	P<0.05
% of patients continued ATM after BC result	56	9.1%	557	95.5%	P<0.05
Days of ATM (mean±SD)	472	9.0±6.1	583	14.8±5.3	P<0.05
Duration ATM after BC results (median [IQR])	56	7 [3-14]	56	12 [7-21]	P<0.05
Mortality	156	22.9%	211	36.2%	P<0.05
Length-of-stay (mean±SD)	682	26.7±53.4	583	31.2±38.1	P=0.10

**SD:** standard deviation; **ATM:** antimicrobial; **BC:** blood culture; **IQR:** interquartile range.

Among the CNS-C samples, 365 (53.5%) were from male patients and 317 (46.5%) were from female patients, with a mean age of 65.1±16.1 years. The mean LOS was 26.7±53.5 days, and the mortality rate was 22.9% (Table 1). The LOS in patients with contamination was 26.7±53.4 and 31.2±38.1 in patients with BSI (P < 0.05). 

Blood cultures with CNS-C were collected from all sectors of the institution, with the highest frequency of samples originating from the wards (55%), followed by the emergency department (39%) and the intensive care unit (16%).

Of all patients with CNS-C, approximately 69.6% (n = 474) initiated ATM immediately after blood collection. The most commonly used ATM was piperacillin/tazobactam (29.1%), followed by ampicillin/sulbactam (26.5%). The distribution of ATM after blood culture collection is shown in Table 2. Only 10.2% of the patients received coverage for MRSA (linezolid, teicoplanin, vancomycin, or daptomycin).

**TABLE 2:** Initial Antibiotic Therapy Choice after Blood Culture Sampling.

Antibiotic	N	%
Aminoglycoside	4	0,8
Penicillins	12	2,6
BL/IBL	322	67,8
Third generation cephalosporin		
Cefepime	12	2,5
Quinolones	12	2,5
Carpabenems	48	10,1
Anti-MRSA drugs	30	6,4
BL/IBL +anti-MRSA drugs	8	1,7
Third generation cephalosporin + anti-MRSA drugs	10	2,1

**BL/IBL:** beta-lactam + beta-lactamase inhibitor; **MRSA:** methicillin-resistant *Staphylococcus aureus*.

Among patients with blood cultures showing CNS-C who initiated ATM ( 69.6%), 9.1% (56 of 682) continued or even changed the ATM regimen after the result was compatible with contamination. In contrast, 95.5% of patients with BSI continued antibiotic therapy (P<0.05). The median duration of ATM use after the culture result was 7 days (IQR 3 - 14) in contamination, and 12 [7-21] in the group of BSI (P<0.05). Although the remaining 418 patients used ATMs but discontinued them after the blood culture results were obtained, ATM was used for an average of 6.1±0.9 days. This was attributed to the delay in releasing the complete results to the medical team.

**FIGURE 2:**
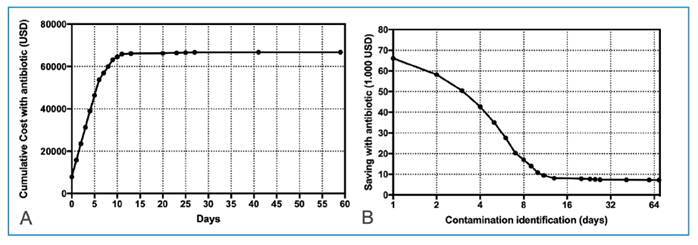
**(A)** Cumulative antimicrobial costs according with the antibiotic duration of the therapy. **(B)** it is an inverse interpretation of potential cost reduction according with antimicrobial stop used for coagulase-negative staphylococci contaminant.

The costs associated with ATMs accumulated according to the duration of use, reaching a total of USD 73,970 during the study period, averaging USD 156 per patient. Costs related to unnecessary identification and susceptibility testing amounted to USD 9,940 during the study period, or USD 20 per patient. Figure 2A depicts the cumulative ATM costs. As shown in Figure 2B, a reverse simulation of the cumulative costs was performed, considering the time of identification of CNS-C. In this study, the average time for the final culture result ranged from 5 to 7 days, showing a point of inflection at 5 days when costs exhibited a significant decrease. 

Patients with CNS-C had a mean LOS of 26.7±53.5 days, compared to 16.5±26.1 days for patients with negative blood cultures, representing a difference of 10.2 days. While this difference cannot be directly attributed solely to contamination owing to possible confounding factors and the high variability in the data (as shown by the large standard deviations), it warrants consideration in the economic impact analysis. If we consider this difference in hospitalization time, with a daily hospital cost of USD 556.80, the potential additional cost would be USD 3,873,323.52 annually for the 682 patients with CNS-C. However, this figure should be interpreted with caution, as the extended length of stay may be influenced by multiple factors beyond blood culture contamination, such as underlying conditions, comorbidities, and other clinical variables not accounted for in this analysis.

**FIGURE 3:**
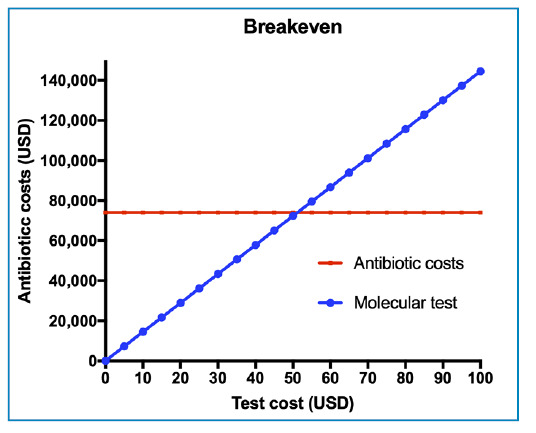
A breakeven between antimicrobial cost (axis y) and possible diagnostic tests for identification of coagulase-negative staphylococci contaminant (axis x). The crosspoint between blue and red line define the maximum cost of the test to be considered cost-effective.

When assessing rapid tests for CNSC identification, such as a direct molecular blood test and a bacterial panel for positive blood cultures with direct identification from the blood culture bottle using matrix-assisted laser desorption/ionization time-of-flight (MALDI-TOF), the equilibrium cost would be USD 50 (Figure 3). The curve illustrates a red line represents the additional costs associated with antibiotics, whereas the blue line depicts the cost of any intervention that has the potential to reduce contamination. On the blue curve, any value to the left of the intersection point between the lines represents a cost-minimizing-or potentially cost-effective-intervention. Whether it qualifies as cost-effective depends on the specific intervention evaluated, but all interventions would be potentially cost-effective, considering the possibility of a reduced LOS.

## DISCUSSION

BSIs are extremely common in the hospital environment, represent a serious complication of the infectious process in critically ill patients, and are associated with high mortality rates and prolonged hospitalization times, making blood culture a test of significant predictive value for infection^14^. Nevertheless, blood culture contamination is a major concern for the correct treatment of BSI. Contamination by coagulase-negative staphylococci in this study was 7% higher in male patients, similar to findings of other studies^15^. Yussuf et al. (2021) reported a larger difference (39%)^16^. In our study, the mean patient age was 65.1 years. Klucher et al. (2022) compared cases of contamination by coagulase-negative staphylococci with control cases and found a significant increase in patient age, which may be a predictor of possible contamination because this type of patient typically has thinner, collapsed, or fragile veins^17^. This characteristic was also related to other associated factors, such as black race, high body mass index, and comorbidities such as congestive heart failure, paralysis, and chronic obstructive pulmonary disease^17^.

In the present study, it was observed that the LOS of patients with false-positive blood cultures was 26.7±53.5 days, while patients with negative cultures had a mean LOS of 16.5±26.1 days, showing a difference of 10.2 days. As expected, patients with true BSI had the maximum LOS (31.2±38.1 days). Although our findings aligned with the logical progression of severity (negative < contamination < true BSI), some studies have found different patterns. Klucher et al. reported in contaminated blood culture patients staying hospitalized for only an additional 1.3 days compared to negative cultures¹⁷. The high standard deviations in our length of stay data indicate substantial variability, and the direct causality between contamination and extended hospitalization cannot be definitively established, as other clinical factors may have influenced the length of stay. Nevertheless, even if only a portion of these additional days was attributable to contamination, the financial impact would still be significant. This highlights the importance of implementing measures to reduce contamination rates, not only for their direct costs (antimicrobials and laboratory tests), but also for their potential contribution to extended hospitalizations^17^.

Sample contamination was found in 9.8% in our study, whereas the Clinical Laboratory Standard Institute (CLSI) recommends a blood culture contamination rate of <3%. However, most institutions fail to meet this target, with contamination rates of 2%-10%^2^. Additionally, among the positive samples (1445), 794 were CNS, representing a contamination rate of 55% among positive cultures, with similar values found in other studies^18^. Patients with difficult venous puncture had higher contamination rates, and certain sepsis protocols, while aiding in antibiotic timing, increased the risk of contamination, posing a concern, given that these patients are potential candidates for septicemia^17^. Studies suggest that practical changes with new interventions and cultural shifts within institutions are necessary to reduce potential contaminations^17^. A solution to reducing contamination occurrences could be the use of a diversion device, which removes 1.5-2.0 mL of blood and contaminants in a separate chamber before collecting blood for culture, particularly in emergency units^19^ ^-^ ^23^.

As analyzed, 69% (475) of cases initiated ATM at the time of blood culture collection. Of these, 9% changed or maintained ATM based on the contaminated blood sample results. In a study by Silbert et al., 61% of blood cultures positive for CNS were considered contaminated, and 32% of these received ATMs based on organism resistance^24^ ^,^ ^25^. The duration of antimicrobial use increased by 9.35 days in patients who changed their regimen after the blood culture results. Klucher et al. (2022) supported this finding, showing an increase in hospitalization time by 6.2 days^17^. A recent study noted a significant reduction in antibiotic days in cases where rapid molecular diagnostic tests were used because of the much lower contamination rate associated with this methodology^26^. It is worth noting that the real need for ATMs in episodes of low-risk BSI is still uncertain^27^ ^,^ ^28^, raising serious questions about the importance of medical clarification at the time of blood culture analysis as well as antimicrobial therapy expertise, as inappropriate conduct exposes patients to improper medication use and burdens the healthcare system. Therefore, institutions must implement rigorous ATM-management practices in institutions^29^ ^-^ ^32^.

In a previous study, reducing the contamination rate lead to an average savings of USD 1,100,214.85 over a year, and the cost of a contaminated culture examination reached USD 10,000 due to potential complications from antibiotic therapy^23^. The total laboratory cost can average 134,018 euros^18^. In another study, Bates et al. reported a 20% increase in laboratory costs and 39% increase in the cost of antimicrobial use in isolation^7^. Gander et al. showed that contaminated blood cultures increased patient expenses by 47% compared to those of patients without laboratory evidence of bacteremia, a result similar to that reported by Bates et al^3^ ^,^ ^7^. Geisler et al. estimated that the aggregate cost of a contaminated blood culture is USD 6,464; 79% of this was associated with an increase in LOS and the value associated with the unnecessary use of antimicrobials^33^.

In our study, antibiotic-related costs were lower than those reported in other publications from developed countries. In Brazil, antibiotic costs were lower because of the use of generics, old antibiotics, and difficulties in using new drugs. Nevertheless, for the public healthcare system in a developing country, these values have a significant impact on reallocating resources to improve diagnosis. In this context, an analysis of implementing technologies to reduce blood culture contamination may not only impact antibiotic reduction, but also reduce prescription errors, earlier de-escalation, and decrease bacterial resistance, which is higher than that in European and American countries^30^ ^,^ ^34^ ^-^ ^38^.

One study showed the financial impact of positive blood cultures for the CNS, with an average cost of USD 7,594. Studies have estimated that false positive CNS blood cultures have a significant impact on hospitalization days (5.4 days) and laboratory and pharmaceutical costs (USD 7,502 at the time of the study), emphasizing the need to standardize blood culture collection. With an increase in the number of hospitalization days, the annual costs were USD 1,905,572^1^. In a study by Klucher et al. (2022), patients with blood culture contamination incurred an additional USD 7,132 in total hospitalization costs, similar to a previous study^17^ ^,^ ^27^.

To reduce unnecessary treatments for blood culture contamination, rapid pathogen identification is crucial, regardless of its rate. Technological advancements have enabled clinical microbiology laboratories to utilize rapid microorganism identification methods, particularly mass spectrometry with MALDI-TOF. The success of this technology is attributed to the broad spectrum of organisms it can identify, the speed (in minutes) at which the results are obtained, the precision and reproducibility of the method, and its cost-effectiveness^39^. However, advances in molecular methods have introduced equipment capable of identifying the different microorganisms present in a sample using real-time polymerase chain reaction (PCR) multiplex technique^40^ ^-^ ^42^. In addition to clinical laboratories and public health reference laboratories, investigations of hard-to-identify microorganisms can be conducted using molecular technology, such as sequencing of the 16S rDNA gene, which is representative of prokaryotic organisms^43^. In our breakeven analysis, considering the local reality, implementing rapid molecular tests would require testing all positive blood cultures (1,452 samples) at a maximum cost of USD 50 per test, resulting in an annual implementation cost of USD 72,600. A direct financial comparison with the current costs of unnecessary antimicrobials (USD 73,970) and microbiological tests (USD 9,940) would show a modest annual benefit of USD 11,310. However, considering that patients with CNS-C stayed an average of 10.2 days longer than those with negative cultures, with a daily hospital cost of USD 556.80, the potential impact became substantially larger. Even if only a fraction of these additional hospitalization days (potentially costing up to USD 3.87 million annually) were attributable to contamination, the financial benefit of rapid identification would be significant. Furthermore, rapid diagnostic methods can provide additional clinical benefits, including faster patient management decisions, reduced unnecessary antibiotic exposure, associated risks of bacterial resistance, and overall improvement in the quality of care. In the breakeven analysis, we can also consider interventions beyond rapid testing, such as preventive measures, including closed blood collection kits, improved antisepsis, and staff training. These strategies are low cost, possibly below USD 50, remaining to the left of the breakeven point on the graph.

In addition to rapid diagnostic methods, it is essential to highlight that the primary and most cost-effective strategy to prevent blood culture contamination remains the proper adherence to hand hygiene protocols and rigorous patient skin disinfection before sample collection. These fundamental practices recommended by international guidelines are especially critical in low- and middle-income countries (LMICs), where access to advanced molecular diagnostics may be limited. Ensuring proper training of healthcare professionals, routine auditing, and adherence to standardized collection techniques can significantly reduce contamination rates. Although technological solutions offer promising results, emphasizing basic infection control measures remains the cornerstone for effective and sustainable contamination prevention in resource-constrained settings.

In resource-limited settings, this comprehensive analysis of both direct and indirect costs strongly supports the implementation of rapid diagnostic methods, and makes it possible to maintain cost maintenance to reduce the unnecessary use of antibiotics. However, the impact here not only considers cost minimization but also cost benefits because of the risk of unnecessary exposure to antimicrobials, fewer infusion adverse events, antimicrobial resistance, hospitalization time, and even mortality^44^ ^-^ ^50^.

The results of our study should be interpreted with caution, as it was a retrospective study based on a cost analysis simulation, which may not accurately reflect real-world outcomes due to less-than-optimal adherence by the medical team. Additionally, we were limited to an institution with a high rate of blood culture contamination. More specific clinical data on treatments, concomitant infections, and reasons for treatment considered unnecessary but directed towards another focus not described in the electronic medical records may introduce information bias.

In conclusion, blood culture contamination leads to unnecessary antibiotic use, resulting in a high cost burden for hospitals. The breakeven analysis indicates that investments up to USD 50 per sample would be cost-effective in this setting.
